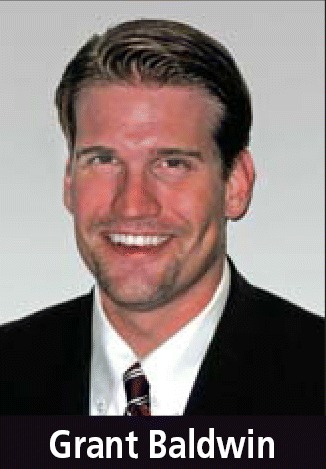# Guest Editorial: Environmental Health and Hurricane Katrina

**DOI:** 10.1289/ehp.114-a12

**Published:** 2006-01

**Authors:** Henry Falk, Grant Baldwin

**Affiliations:** Coordinating Center for Environmental Health and Injury Prevention, Centers for Disease Control and Prevention, Atlanta, Georgia, E-mail: hxf1@cdc.gov

Hurricane Katrina caused enormous physical destruction, environmental degradation, and human misery (Travis 2005). Full remediation will take years, and many decisions that are fundamental to the restoration and rejuvenation of the Gulf Coast are yet to be made. The challenges for New Orleans, Louisiana, are particularly complex.

Criticism of the disaster response and preparedness effort was swift and intense (Anonymous 2005; Coghlan and Mullins 2005). Who will forget the searing photographs of stranded and desperate New Orleanians in the days after the city was flooded? Lessons abound and will undoubtedly inform future disaster planning at all levels for many years (Nieburg et al. 2005).

Compounding the devastation in New Orleans was the near total disruption of the public health and medical infrastructure. Although federal disaster preparedness plans include provisions of surge capacity through Disaster Medical Assistance Teams (DMATS) and other resources, extraordinary and often improvised measures were needed in New Orleans and in numerous shelters and points of refuge to cope with the scale of the displaced population. State and local governments, the U.S. Public Health Service within the Department of Health and Human Services (HHS), other federal agencies, academic institutions, private and nonprofit organizations, and an outpouring from the lay public contributed enormously to the immediate postevent response. The acts of heroism and dedication among the public health and medical communities during and immediately after hurricane Katrina are numerous (Berggren 2005; Raggi and Raggi 2005; Schwartz 2005).

An early report from a Centers for Disease Control and Prevention (CDC)/U.S. Environmental Protection Agency (EPA) team provided an initial overview of the environmental health issues in New Orleans in relation to safe rehabitation of the area (CDC/U.S. EPA 2005). Issues related to housing, debris removal, toxic chemicals, sewage treatment, safe drinking water, and occupational health headed the list. Although essential infrastructure and supporting services have been restored in some areas of New Orleans, this is not yet the situation in many of the hardest hit areas of the city.

Individually, these are all very complex and difficult problems. The housing stock is being systematically evaluated for structural integrity and viability, and a significant number will not be viable. The decision-making process for the housing stock will be affected by the financial impact of rebuilding requirements, such as the need to be above the flood plain, and by city-wide plans for low-lying neighborhoods.

Mold is ubiquitous and is present in almost unprecedented quantities in New Orleans (CDC Mold Workgroup 2005). This poses a critical health risk to returnees and particularly to sensitive populations. City government and health authorities responded by providing guidance and a wide array of educational materials to returning homeowners and physicians (CDC 2005; U.S. EPA 2005). Despite the emphasis on prevention and surveillance, concerns about mold-related symptoms have surfaced (“Katrina Cough”) and need sustained attention to assure that unexpected problems are identified and revised prevention messages are disseminated as quickly as possible.

The scope of the debris removal is so large that it has forced officials to consider the use of incineration and other volume-reduction strategies, such as grinding, as well as creating new landfills or reopening existing landfills. Environmental health authorities have emphasized best practices for preventing exposure to hazardous substances in sediment while conducting extensive sampling and focusing on localized hot spots of potentially significant exposure (Pardue et al. 2005; U.S. EPA 2005). Environmental groups have highlighted the hot spots and expressed concern about prolonged, close contact with sediments.

The drinking-water and sewage systems are also not fully operational. The damage to the central plants and to the extensive distribution and collection systems for the respective drinking-water and sewage systems are being systematically repaired but will take many more months for completion. Moreover, upgrades to these older systems need to be considered.

Successful recovery will require the breadth of vision and wisdom to link multiple environmental health solutions to the many broad decisions being made by governments, communities, industry, and other key stakeholders. The simultaneous restoration of all of these individual environmental health services presents a unique situation and governmental challenge. Clear and coordinated communication and outreach across stakeholders will play an important role in the recovery.

Disruption in medical care, loss of relatives and friends, stress and mental health concerns, ad hoc living arrangements, separation from home and community, financial ruin, and many other factors contribute to the difficulty in resuming “normal life” (Voelker 2005). Those with individual resources and professional skills will be more able to either return and rebuild or to integrate elsewhere, in spite of the challenges. The greatest concern will be for those with limited means and ability to start over on their own; who will have been in constant flux from shelters to hotels to trailer parks; and who will be facing an uncertain future. The individuals in the lowest-lying and most flood-prone and vulnerable areas are often those with the greatest difficulties in restoring, rebuilding, and resuming their lives; they will also have the greatest need for governmental and other assistance. Hurricane Katrina has so visibly reinforced the impact and need for addressing health disparities (Atkins and Moy 2005).

A fundamental question facing New Orleans, and influencing many other decisions, is how to make the city safe from future hurricanes. There is now intense focus on the levee system for New Orleans, and a number of groups have recently raised concerns about design flaws and other lapses in this system (Kintisch 2005; Seed et al. 2005). Can a better system be designed? What degree of protection will be provided (for a Category 3, 4, or 5 hurricane)? Can the low-lying parts of New Orleans be adequately protected? Are the financial resources and management wherewithal available to accomplish this in a timely manner? If not, what next? If yes, will these solutions work for the long term, given the predictions of sea level change by global climate theories? Or, more directly, will other major construction projects also be necessary to increase silt and sediment deposits for restoration of coastal wetlands and marshes (Stokstad 2005)? All of these are very difficult, but necessary, questions to answer.

There is a strong need and desire to maintain the essential character of New Orleans and other devastated Gulf Coast areas as they are restored and rebuilt. At the same time many environmental health proponents will also see the unique opportunities for applying the principles of smart growth to the rebuilt environment and wisely balancing out the old and the new.

This unprecedented disaster poses many difficult environmental health challenges. The skills and talents of many in our professional community will contribute to the recovery of New Orleans and the Gulf Coast region.

## Figures and Tables

**Figure:**
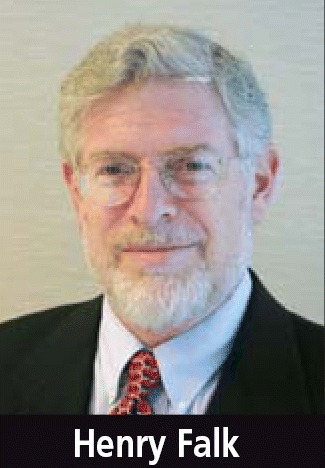


**Figure:**